# Supplementary material for: Chromothripsis during telomere crisis is independent of NHEJ, and consistent with a replicative origin
Source: Genome Res. 2019 May;29(5):737–49. doi: 10.1101/gr.240705.118 (PMC6499312; doi:10.1101/gr.240705.118)
Supplement: Supplemental Material [file supp_gr.240705.118_Supplemental_file_1.zip › contigs/annotated_contigs/DB113/contig.2.DB113_length_710_mean_cov_8.02394366197.docx]

**DB113_length_710_mean_cov_8.02394366197**

```
AAGACAGAATTCTATCTCAAATCTACCTTCAGTATAATGAGACATTAGATGTTAATACAGAATAACATACCCTTTAAGACAGAGGGCAA
 >chr11:103255465-103255745 - E=1e-156
ATTAAAGGCATTTTCAGATAAAAACTCAAGAGTTCACAACCAAAAGATACTTTCTACACTATAAGCAGATGCAAAGTGAAACCAAACAG

AATGTCTGAGATACAGGAAGAAATAAAGAACAATACAAGTGATAAACATGGGGCAAATCCAAATAAGCATTATGTACATGACTACAACA

ACAAGAAAGA|CAG|TGCTTTTATTAAGATTTTTAAAAGGAGAGGCCGGGCATGGTGGTTCACGTCTGTAATCCCAGCACTTTGGGAGG
           >chr11:103254125-103254532 - E=5e-215
CTGAGGCGGGCAGATCATGAGGTCAGGAGATGGAGACCATCCTGGCTAACATGGTGAAACCCCGTCCCTACTAAAAATACAAAAAAATT

AGCTGGGCGTGGTGGTGGGTACCTGTGGTCTCAGCTACTCGGGAGGCTGAGGCAGGAGAATGGCGTGAACGCGGGAGGCAGAGCTTGCA

GTGAGCCAAGATTGCGCCACTGCACTCCAGCCTGGACAATAGAGCGAGACTCCATCTCAAAAAAAAAAATAAAGATTAAAAAGGAGAAA

GAATTACAAAACAAACCACCCAAATCAAAAAGCAAAACATTAGAGAAAAAAACCAATAAAG|ACCGTGCAATCGAAAAGGAAAATCCTA

T
```
